# Enjoyment of exercise moderates the impact of a school-based physical activity intervention

**DOI:** 10.1186/1479-5868-8-64

**Published:** 2011-06-20

**Authors:** Margaret Schneider, Dan M Cooper

**Affiliations:** 1 Department of Planning, Policy, and Design, University of California at Irvine, California, USA; 2 Department of Pediatrics, University of California at Irvine, Orange, California, USA

## Abstract

**Background:**

A school-based physical activity intervention designed to encourage adolescent girls to be more active was more effective for some participants than for others. We examined whether baseline enjoyment of exercise moderated response to the intervention.

**Methods:**

Adolescent girls with a low level of baseline activity who participated in a controlled trial of an intervention to promote increased physical activity participation (*n *= 122) self-reported their enjoyment of exercise and physical activity participation at baseline, mid-way through the intervention, and at the end of the 9-month intervention period. At all three time points, participants also underwent assessments of cardiovascular fitness (VO_2_peak) and body composition (percent body fat). Repeated measures analysis of variance examined the relationship of baseline enjoyment to change in physical activity, cardiovascular fitness, body composition and enjoyment of exercise.

**Results:**

A significant three-way interaction between time, baseline enjoyment, and group assignment (p < .01) showed that baseline enjoyment moderated the effect of the intervention on vigorous activity. Within the intervention group, girls with low enjoyment of exercise at baseline increased vigorous activity from pre-to post-intervention, and girls with high baseline enjoyment of exercise showed no pre-post change in vigorous activity. No differences emerged in the comparison group between low-and high-enjoyment girls.

**Conclusion:**

Adolescent girls responded differently to a physical activity promotion intervention depending on their baseline levels of exercise enjoyment. Girls with low enjoyment of exercise may benefit most from a physical-education based intervention to increase physical activity that targets identified barriers to physical activity among low-active adolescent girls.

## Background

Evidence for the health-enhancing effects of physical activity continues to accrue, with many studies demonstrating the detrimental effects of inactivity [1,2]; yet, within the past century there has been a distressing increase in the prevalence of physical *in*activity. In particular, rates of activity decline precipitously during adolescence [3], thus making the promotion of physical activity in this age group a public health priority [4]. The precise proportion of adolescents who fail to meet the current recommendations for physical activity (at least 60 minutes of moderate-to-vigorous activity [MVPA] on most days) is difficult to quantify, since estimates vary widely depending on the method used to collect the data, but estimates are consistently below 50% [5-7]. Clearly, adolescence is a critical period for promoting physical activity.

Physical Education (PE) classes in schools have been touted as the optimal avenue for promoting adolescent physical activity [8] in the form of exercise, which is a subcategory of physical activity that refers to planned activity undertaken specifically for the purposes of fitness and/or recreation. The current evidence does suggest that PE-based interventions have a positive impact on adolescent physical activity [9]; yet, on closer evaluation, the effects are modest and short-lived [10-12]. Interestingly, it is not usually possible to detect whether certain subgroups may have responded well to the interventions, and others less well, thus lowering the mean response. In work with low-active adolescents, a PE-based intervention increased out-of-school physical activity [13], but the variability in responses was considerable [14], suggesting that the intervention was more effective for some individuals than others.

Over the course of the last decade, a number of randomized controlled trials have evaluated the impact of PE-based interventions on adolescent girls [15-18], who are of particular interest owing to their lower participation in physical activity compared to boys at all ages [3]. The largest of these studies was the Trial of Activity for Adolescent Girls (TAAG; [16]), in which 18 intervention schools were compared to 18 control schools across a 2-year intervention that targeted physical education within a comprehensive intervention that encompassed community agencies and a social marketing effort. The findings from TAAG suggest that the intervention may have been somewhat effective in preventing a decline in activity among girls in the intervention group, but less effective for increasing levels of physical activity.

A similar approach to increasing adolescent girls' activity levels was taken in the Lifestyle Education for Activity Program (LEAP; [15]), which supported a PE-based program with components targeting health education, school environment, school health services, faculty/staff health promotion, and family/community involvement. Girls at 12 intervention schools were compared to girls at 12 control schools before and after a 1-year intervention. At the end of the year, 45% of the intervention girls and 36% of control girls reported engaging in 30 minutes of vigorous activity over the past 3 days. While evidence of intervention success, these data also demonstrate that fully 55% of the intervention girls did not increase their activity levels over the course of the year. Both TAAG and the LEAP study highlight the need to identify characteristics of adolescent girls that may predispose them to respond favorably to a school-based physical activity intervention.

Analysis of the available evidence raises the possibility that the modest impact of school-based interventions for mobilizing the majority of adolescents may be rooted in an insufficient attention to strategies that cultivate adolescents' enjoyment of physical activity and a corresponding failure to motivate the least-motivated youth. Among adults, certain population subgroups respond better to particular intervention strategies, with those who are more active at baseline responding to a traditional intensity-based approach, and those who are less active at baseline responding to an approach that encourages more moderate, lifestyle-based activity [19]. Within school-based settings, greater success in promoting out-of-school physical activity accrues when the intervention is targeted to low-active adolescents and/or employs strategies designed to enhance adolescents' enjoyment of physical activity [11,13,20].

Hedonic theory posits that people will be motivated to engage in behaviors that bring them pleasure and avoid activities that are accompanied by feelings of displeasure [21,22]. Consistent with this theory, a positive affective response to exercise correlates with physical activity among both adults [23] and adolescents [24]. Similarly, students who report enjoying PE more engage in greater physical activity outside of school [25]. Studies that have found a positive association between habitual exercise participation and the affective response to an acute exercise task typically conclude that regular exercise cultivates enjoyment of physical activity [26]. Alternatively, it is plausible that individuals differ in their innate predisposition to experience positive affect in conjunction with exercise, and that differences in the affective response to exercise between low-active and active persons is a reflection of this underlying predisposition.

The hypothesis that individuals differ in their innate predisposition to enjoy exercising might help to explain some of the resistance to change in activity evidenced by adolescent programs targeting physical activity. Among adolescents who do not enjoy exercising, an intervention that is designed to encourage them to be more active may only serve to confirm their dislike of activity. In contrast, among adolescents who do enjoy exercising, the energy that is injected into a school PE program during a research-based intervention may facilitate their latent desire to be more active, and inspire them to generalize this heightened activity outside the school environment. Alternatively, PE-based programs designed to increase adolescents' enjoyment of activity may not have much impact on those who already enjoy exercising, and may be most effective among adolescents who report a low level of enjoyment of exercise. Evidence derived in a laboratory setting suggests that the proportion of adolescents who experience a positive affective response to hard exercise is relatively small [24]. Knowing whether PE-based interventions are most effective within the relatively small proportion of the population who already enjoy exercising or among the much larger proportion of the population who report low exercise enjoyment would provide valuable information that schools could consider when weighing the return on investment for enhanced PE programs.

A previous report on Project Fitness and Bone (FAB) showed that the school-based intervention increased out-of-school vigorous activity and cardiovascular fitness within the intervention group [13]. However, responses to the intervention were highly variable [14], with some individuals in the intervention group actually declining in activity. The present study sought to determine whether Project FAB had a differential impact on participants depending on their baseline enjoyment of exercise. We tested two alternative hypotheses: 1) girls who reported higher enjoyment of exercise at baseline would respond more positively to the intervention, as they would be more receptive to the new opportunities and encouragement for being physically active; 2) girls who reported lower enjoyment of exercise at baseline would show the greatest improvement in physical activity, as they had the most to gain from increasing their level of exercise enjoyment.

## Methods

### Study Design

A complete description of the study design and procedures is provided elsewhere [13]. This study reports data from the 122 girls who completed the study. Participants were recruited using direct mailings, flyers distributed at school, and classroom presentations. Girls were eligible to participate if they did not participate on any team or club sports. Additionally, girls were excluded from the study if they scored above age-specific 75^th ^percentile for cardiovascular fitness at baseline.

Assessments were conducted at baseline (summer; June-August), semester one (the end of fall semester; November and December) and semester two (the end of spring semester; April and May). All assessments described below were obtained at all three time points. The study protocol was reviewed and approved by the university's Institutional Review Board, and all participants and a parent or guardian provided written informed consent.

### Intervention

The intervention was administered at a public school in a middle-class suburb during the normal school day. The intervention goal was to increase students' levels of physical activity through supervised in-class activity, health education, and internet-based self-monitoring. The class met five days per week for 60 minutes each day (approximately 40 minutes of activity time). One day per week was devoted to an educational discussion related to the health benefits of exercise and strategies for adopting an active lifestyle. Elements of the intervention were included to make the class appealing for low-active females, including the following: participants were exempted from the usual timed mile run requirement in PE; participants were excused from wearing uniforms during PE; activities were changed more frequently than the typical PE class; participants had input into the choice of activities during the course of the year; and activities were modified to be appropriate for individuals with lower levels of fitness (e.g., half-court basketball instead of full-court basketball). A detailed description of the intervention has been provided elsewhere [13].

### Measures

#### Cardiovascular fitness

Cardiovascular fitness was obtained through a ramp-type progressive exercise test on an electronically-braked cycle ergometer. Participants were encouraged to maintain a pedaling rate of 70 rev min^-1 ^during the test phase of the protocol. The ramp power output increased continuously until participants reached voluntary fatigue. The test portion of the protocol lasted between 8-12 minutes. Each test was followed by an appropriate cool down period. Peak oxygen consumption (VO_2_peak in L min^-1 ^and VO_2_peak in ml/min/kg) was obtained using the SensorMedics Vmax 229 metabolic cart (Yorba Linda, CA), through a method designed for children and adolescents[27]. Gas exchange was measured breath-by-breath throughout the exercise protocol [28].

#### Body composition

Percent body fat was assessed by dual x-ray absorptiometry (DXA) using a hologic QDR *4500 densitometer (Hologic, Inc. Bedford, MA). Scans were performed by a licensed x-ray technician and analyzed using pediatric software. Participants were scanned in a hospital patient gown while lying flat on their backs. On each day of testing, the DEXA machine was calibrated using the procedures provided by the manufacturer. Height was measured to the nearest 0.01 cm using a stadiometer and weight was measured to the nearest 0.1 kg using a calibrated scale.

#### Physical activity recall

Self-reported physical activity was measured using a 3-Day Physical Activity Recall (3DPAR) validated by Motl, Dishman, Dowda, and Pate[29]. Activities were converted into Metabolic Equivalents (METs) using the compendium published by Ainsworth et al.,[30] and grouped to calculate the average daily minutes spent engaged in MVPA (3 METs and above) and vigorous physical activity (6 METs and above). The 3DPAR was always administered on a Tuesday, and students in the intervention group were not active during the PE period on Mondays (these days were reserved for the educational portion of the intervention). Consequently, the activity reported on the 3DPAR was limited to activity that occurred outside the school day.

#### Enjoyment of Exercise

The 18-item PACES instrument (Physical Activity Enjoyment Scale) developed and validated by Kendzierski and DeCarlo [31] was employed to assess exercise enjoyment. Each item was presented as a semantic differential (e.g., "I enjoy it" to "I hate it"). Respondents circled the number (on a scale of 1 to 5) corresponding to the degree of affinity for one of the anchors. Cronbach's alpha in this study was .91.

### Data analyses

All variables were examined for outliers and non-normal distributions. The vigorous activity variable was highly skewed, and so was transformed using the square root procedure prior to analyses. To investigate the potential moderating influence of baseline exercise enjoyment on the intervention impact (MVPA, vigorous activity, VO2peak, and percent body fat), we employed a series of 3 (Time: baseline, semester one, semester two) × 2 (Group: intervention vs. Comparison) × 2 (Baseline Enjoyment: high vs. low) mixed design analyses of variance (ANOVAs). Potential covariates included baseline cardiovascular fitness (VO2peak ml/kg/min), and percent body fat (for analyses predicting activity variables) and race (White/non-White). The intervention group included a greater proportion of non-Hispanic Whites (68% vs. 49%; χ^2 ^(df = 1) = 4.03, P < .05), but there was no association between ethnicity and the outcome variables, nor did including it as a covariate modify the results of the analyses, so it was not included in the analyses. Similarly, VO_2_peak and percent body fat were considered and rejected as covariates. Separate analyses were conducted using vigorous activity, MVPA, cardiovascular fitness, and percent body fat as the dependent variables. An additional mixed design ANOVA examined the potential moderating effect of baseline enjoyment on change in exercise enjoyment over time. When the assumption of sphericity was violated, the Huyn-Feldt adjustment was used.

## Results

### Descriptive statistics

The average age of participants was 15.04 (SD = .78) years; all girls were enrolled in the 9^th ^grade during the intervention. Most of the study participants were non-Hispanic Whites (57%), with the remainder Latina (20%), Asian (17%) or other/mixed (4%). As expected, given the study inclusion criteria, girls were above the median for BMI percentile (66.20 [27.09]), though activity levels were not as low as might be expected; mean daily vigorous activity was approximately 20 minutes per day (19.34 [30.68]), and daily MVPA was about 80.65 [74.59] minutes per day. The relatively high activity probably reflects the timing of the baseline data collection, which occurred during summer vacation. Mean VO_2_peak was 23.44 [4.51] mL/min/kg, which falls in the "low" fitness range compared to national averages for this age group [32].

Girls within each group (intervention and comparison) were clustered into two groups based on their scores being above or below the median for enjoyment of exercise (3.44 on a scale from 1 to 5). At baseline, intervention and comparison girls were comparable in age, height, weight, body mass index and percent body fat [13]. In t-test comparisons between the low-and high-enjoyment girls within each group (intervention and comparison), the only significant difference that emerged was between low-and high-enjoyment girls in the intervention group. Girls in the intervention group who reported having a higher level of enjoyment for exercise engaged in more minutes per day of vigorous activity as compared to their compatriots who reported less enjoyment of exercise (p < .05).

### Test for moderation of intervention impact by baseline enjoyment

The association between baseline enjoyment and change in vigorous activity was assessed using a 3 (Time: baseline, semester one, semester two) × 2 (Group: intervention vs. comparison) × 2 (Baseline Enjoyment: high vs. low) mixed design ANOVA. There was a main effect of time (*F*(2, 117) = 3.21, *P *< .05, partial eta-squared = .05) and a significant three-way interaction between time, intervention group and baseline enjoyment (*F*(2, 117) = 6.67, *P *< .01, partial eta-squared = .10). Post-hoc t-tests indicated that the only group that showed a significant improvement in vigorous activity over time was the low-enjoyment intervention group (*T *= -2.67, *P *< .05). Within this group, participation in vigorous activity increased from a mean of approximately 13 minutes per day to a mean of 18 minutes per day, whereas in the low-enjoyment girls in the comparison group vigorous activity declined from 21 minutes at baseline to approximately 15 minutes after the intervention (see Table 1). Interestingly, within the intervention group, there was a linear pattern of increasing participation over time for girls with low baseline enjoyment, whereas there was a mid-year decline in vigorous activity participation among intervention girls with high baseline exercise enjoyment and among comparison girls with low exercise enjoyment.

**Table 1:** Changes in enjoyment, fitness, and activity over time [M(SD)].

	Intervention Group					
	**Low Enjoyment (*n *= 29)**			**High Enjoyment (*n *= 34)**		
	
	**Baseline**	**Semester 1**	**Semester 2**	**Baseline**	**Semester 1**	**Semester 2**

Enjoyment	2.75 (.46)	3.35 (.67)	3.32 (.79)	3.87 (.38)	3.68 (.60)	3.66 (.78)
VO_2_peak	23.67 (4.40)	23.44 (4.36)	25.45 (5.26)	22.77 (5.16)	23.01 (4.74)	23.11 (3.58)
Vigorous	12.90 (24.65)	14.51 (11.70)	18.06 (11.66)	30.31 (34.49)	11.56 (12.21)	20.62 (13.66)
MVPA	65.16 (69.27)	51.29 (57.66)	50.96 (51.33)	84.68 (85.60)	51.25 (66.02)	89.68 (64.48)
	**Comparison Group**					
	Low Enjoyment (*n *= 31)			High Enjoyment (*n *= 28)		
	Baseline	Semester 1	Semester 2	Baseline	Semester 1	Semester 2

Enjoyment	2.94 (.43)	3.23 (.56)	3.45 (.44)	3.85 (.30)	3.68 (.60)	3.85 (.53)
VO_2_peak	22.99 (4.99)	21.25 (4.12)	22.41 (3.91)	24.50 (2.97)	23.10 (4.02)	23.34 (3.25)
Vigorous	21.29 (32.32)	9.67 (17.22)	14.83 (18.23)	11.78 (27.62)	11.78 (13.34)	12.14 (15.95)
MVPA	69.67 (59.13)	37.41 (45.96)	53.22 (65.69)	105.35 (78.99)	56.42 (65.27)	58.57 (73.52)

*Note*. Non-adjusted means are presented. Enjoyment was assessed on a 5-point scale. VO_2_peak is in ml min^-1 ^kg. Vigorous physical activity and MVPA are in mean minutes per day.

^a ^Vigorous activity was greater at baseline among intervention girls with high, as compared to low exercise enjoyment.

^b ^A significant 3-way interaction between time, group, and baseline enjoyment indicated that baseline enjoyment moderated the effect of the intervention on vigorous activity.

A 3 (Time: baseline, semester one, semester two) × 2 (Group: intervention vs. comparison) × 2 (Baseline Enjoyment: high vs. low) mixed design ANOVA was used to examine the possible moderating effect of baseline enjoyment on the intervention's impact on cardiovascular fitness. There was a main effect of time (*F*(2, 112) = 4.75, *P *< .05, partial eta-squared = .07) and an interaction between time and group (*F*(2, 112) = 6.91, P < .01, partial eta-squared = .02), but no three-way interaction between time, baseline enjoyment and group. An analogous equation was used to test for a moderating effect of baseline enjoyment on the intervention's impact on MVPA. Again, there was a main effect of time (*F*(2, 112) = 13.47, *P *< .001, partial eta-squared = .18) and an interaction between time and group (*F*(2, 112) = 3.23, P < .05, partial eta-squared = .05), but no three-way interaction between time, baseline enjoyment and group. Finally, the potential moderation of the intervention's impact on percent body fat revealed a main effect of time (*F*(2, 117) = 6.10, P < .01, partial eta-squared = .09), but no significant interactions.

### Change in enjoyment of exercise over time

The relative change in enjoyment of exercise over time in the high-enjoyment and low-enjoyment groups was assessed using a 3 (Time: baseline, semester one, semester two) × 2 (Group: intervention vs. comparison) × 2 (Enjoyment: high vs. low) mixed design ANOVA. There was a main effect of time on enjoyment (*F*(2, 116) = 4.25, *P *< .05, partial eta-squared = .06) and a significant interaction between baseline enjoyment and time (*F*(2, 116) = 13.15, *P *< .001, partial eta-squared = .18). Within both the intervention and the comparison groups, enjoyment of exercise increased over time among the girls who began the study with a low level of exercise enjoyment; no increase in exercise enjoyment occurred among the girls who were above the median for exercise enjoyment at baseline.

## Discussion

This study set out to test the hypothesis that baseline enjoyment of exercise would moderate the impact of a school-based physical activity intervention. The results showed that indeed adolescent girls with relatively low self-reported enjoyment of exercise responded differently to the intervention as compared with girls who reported relatively high exercise enjoyment at baseline. The greatest impact of the intervention was observed among the girls with lower enjoyment of exercise at baseline. Among this subgroup of participants, participation in vigorous activity increased, whereas vigorous activity decreased or remained constant within the rest of the subgroups. Moreover, the improvement among the girls in the low-enjoyment group was linear over time, whereas the high-enjoyment intervention group showed a transient decline in vigorous activity at the midpoint of the intervention.

It should be noted that baseline enjoyment did not moderate the impact of the intervention on MVPA, but only on vigorous activity. We posit that the lack of a moderation effect on MVPA may reflect the different motivations that come into play for activities considered moderate as opposed to those considered vigorous. The specific activities listed on the 3DPAR and classified as vigorous include: aerobic dancing, basketball, bicycling, jogging/running, soccer, stationary exercise machines, tennis, and swimming. The additional activities that would be classified as "moderate" and therefore be incorporated into the MVPA assessment include: dancing, walking, volleyball, bowling, cheerleading, and calisthenics. Because we excluded girls involved in organized sports, the moderate-intensity activities that were most likely to be reported within our sample were walking and dancing. Each of these activities tended to occur in conjunction with some special event (e.g., a school dance or party, or a trip to Disneyland), and were not, therefore, a reflection of a motivation to be physically active. In contrast, the activities classified as vigorous tend to be those that require planning and deliberate intention. Thus, whether or not an individual enjoys being active may be more relevant to participation in vigorous activity than to participation in MVPA, at least as assessed in the present study.

The results of these analyses build on and extend the findings from the LEAP study [33], in which enjoyment of exercise was found to mediate the impact of a school-based intervention to promote physical activity among adolescent girls. The data from the LEAP study suggest that by increasing girls' enjoyment of physical activity an intervention might bring about greater activity levels. Our results indicate that this dynamic occurs among girls who enter the intervention with a relatively low level of exercise enjoyment and that girls with a relatively high level of exercise enjoyment do not benefit from the intervention either in terms of gains in exercise enjoyment or in terms of higher levels of activity. The implication of these two findings in concert is that a targeted intervention that increases enjoyment specifically among girls with a low level of exercise enjoyment would likely provide a greater return on investment than a more diffuse program delivered to the general adolescent population.

The finding that vigorous activity participation actually declined at the midpoint of the intervention among the girls who reported high baseline exercise enjoyment is most likely attributable to the timing of the assessments. As noted previously, the baseline assessment was obtained during the summer vacation, when most girls would have plenty of free time to engage in physically active leisure pursuits. In contrast, the assessment at the end of semester one was obtained right in the middle of the school year, when girls would be juggling other time constraints related to school. Because our method of assessing physical activity specifically excluded any activity obtained in PE, the activities reported had to occur during out-of-school time. Thus, it is not surprising that the most active girls in the summer may have experienced a slight decline in activity during the school year. By the end of the year, however, the high-enjoyment girls were reporting levels of activity that approached baseline levels. We speculate that their enduring enjoyment of physical activity enabled them to adapt to the competing time demands of school over the course of the year.

The finding that self-reported enjoyment of exercise increased over time in the girls who reported low enjoyment of exercise at baseline raises several questions. There is a growing literature suggesting that there may be individual differences in the affective response to exercise [34,35] and that these individual differences may be related to levels of physical activity participation [23,24]. Currently, however, there are inadequate longitudinal studies to determine whether these individual differences represent a stable trait or a malleable, experience-dependent characteristic. That is, there is insufficient evidence at this point to demonstrate either that individuals possess a stable affective disposition toward exercise or, alternatively, that individuals' affective response to exercise may be modified by experience. Our findings contribute to this debate, and suggest that it is worth exploring ways to increase exercise enjoyment among low-active adolescent girls.

Our findings would suggest that the affective response to exercise may be modified through experience, as the girls in the low-enjoyment group at baseline clearly increased their reported enjoyment over time commensurate with an increase in vigorous activity participation. However, the type of exercise that girls in this study were asked to do during the study differed substantially from the standard PE class that they would have been exposed to previously. Considerable modifications were made to the typical PE curriculum: girls were exempted from aspects of the PE class that had previously been identified, through focus groups, as aversive to low-active girls (i.e., completing the timed mile run and wearing uniforms); novel activities were added to the curriculum to appeal to girls' ability levels and preferences (e.g., yoga, brisk walking); girls in the class were able to have an influence over the activities that were offered, thus providing them with some degree of control over the activities; and the intervention was offered only to girls who were not active in competitive sports.

All of these innovative elements likely had an impact on the girls' perceived competence for exercise, their sense of autonomy, and the feeling of belonging that comes from participating in an activity within a supportive group setting. In short, the intervention targeted the three psychological needs (competence, autonomy, and relatedness) that have been identified in Self-Determination Theory [36] as factors that contribute to intrinsic motivation (i.e., inherent pleasure in an activity for its own sake). Thus, whereas reported enjoyment of exercise increased within the Project FAB girls who had lower reports of enjoyment at baseline, we do not know whether this level of enjoyment would generalize to other settings, such as within a regular PE class. The shift in enjoyment may have been entirely context-dependent, and there may still be a trait component to the affective response to exercise that would place these girls at higher risk for avoiding activity once they moved on from the special environment established during the study.

Despite the enhanced impact of the intervention on vigorous activity participation among the low-enjoyment girls, we did not find a differential impact of the intervention on cardiovascular fitness or percent body fat. It would appear that the magnitude of the difference in activity was not sufficient to bring about an detectable difference in fitness over 9 months. Visual inspection of the data do show a trend toward improved fitness among the low-enjoyment intervention girls; a trend that is absent among the low-active comparison girls. A larger study might be able to detect a significant difference between these two groups.

Although encouraging, the results of this study are limited by certain aspects of the study design. The measure used to assess enjoyment of exercise is subject to all of the vagaries of self-report instruments, including self-presentation bias. Study participants knew that they were enrolled in a program intended to increase their physical activity, and they formed relationships with the investigators and research assistants over time. There may have been some motivation to answer questions in a progressively more positive fashion over time, although there is no reason to suppose that this effect would be more prominent among the girls who reported low enjoyment of exercise at baseline. The outcome variable, vigorous activity, also was obtained via self-report and may have been subject to recall bias and testing effects. More accurate assessments of activity levels might have been obtained using more objective tools, such as accelerometers. As participants moved through the study and completed the 3DPAR for the second and third times, they may have become more accurate in reporting. If so, one might expect that the report of vigorous activity would have declined over time, since most people tend to over-report their activity levels. However, there is also the possibility that girls over-reported their vigorous activity over time as a result of social desirability influences. Again, however, it is not immediately obvious why this effect would have been greater among the girls who reported enjoying exercise less at baseline.

Future investigations might extend these findings by incorporating a more standardized assessment of exercise-associated affect prior to implementing an intervention. This approach was employed by Williams et al. [23], who obtained a measure of the affective response to exercise during a submaximal cardiovascular fitness test prior to a physical activity intervention among adults. They found that adults with a more positive affective response to the task increased their activity more over time. Their findings, which run counter to the present study results, again suggest that there may be context-dependent elements to the self-report of exercise enjoyment, and that future studies should attempt to tease apart what may be trait and state elements of exercise-associated affect.

## Conclusions

Our findings are encouraging in that they support the utility of PE-based interventions that target low-active adolescent females with the intent of increasing out-of-school activity. The intervention was in fact more effective among girls who reported a low level of exercise enjoyment at baseline, perhaps suggesting that these girls were most responsive to the modifications in the PE environment introduced during the intervention. Our results demonstrate that school-based interventions may have varying impact on different subgroups of participants; these differential impacts should be explored when evaluating the "success" of school-based interventions.

## Competing interests

The authors declare that they have no competing interests.

## Authors' contributions

MS conceived of the study, supervised the intervention and data collection, performed data analyses, and drafted the manuscript. DC contributed to the design of the study and selection of measures, advised on analytical approach, and reviewed and commented on the manuscript. Both authors read and approved the final manuscript.
